# Demographic and Clinical Characteristics of Hospitalized Patients with Type 2 Diabetes Mellitus and Comorbid Parkinson’s Disease in Spain: A Nationwide Observational Study (2017–2023)

**DOI:** 10.3390/jcm14134679

**Published:** 2025-07-02

**Authors:** Víctor Gómez-Mayordomo, Rodrigo Jiménez-García, José J. Zamorano-León, David Carabantes-Alarcón, Andrés Bodas-Pinedo, Valentín Hernández-Barrera, Ana López-de-Andrés, Natividad Cuadrado-Corrales

**Affiliations:** 1 Neurology Department, Vithas Madrid La Milagrosa University Hospital, Institute of Neuroscience, Vithas Hospital Group, 28003 Madrid, Spain; victorantonio.gomez@ext.vithas.es; 2 Department of Public Health and Maternal & Child Health, Faculty of Medicine, Universidad Complutense de Madrid, Instituto de Investigación Sanitaria del Hospital Clínico San Carlos (IdISSC), 28040 Madrid, Spain; josejzam@ucm.es (J.J.Z.-L.); dcaraban@ucm.es (D.C.-A.); abodas@ucm.es (A.B.-P.); mariancu@ucm.es (N.C.-C.); 3 Preventive Medicine and Public Health Teaching and Research Unit, Faculty of Health Sciences, Universidad Rey Juan Carlos, 28922 Madrid, Spain; valentin.hernandez@urjc.es; 4 Department of Public Health and Maternal & Child Health, Faculty of Pharmacy, Universidad Complutense de Madrid, Instituto de Investigación Sanitaria del Hospital Clínico San Carlos (IdISSC), 28040 Madrid, Spain; anailo04@ucm.es

**Keywords:** Parkinson’s disease, Type 2 diabetes mellitus, comorbidity, COVID-19, in-hospital mortality, hospitalization, sex

## Abstract

**Background/Objectives:** Type 2 diabetes mellitus (T2DM) and Parkinson’s disease (PD) are two highly prevalent chronic conditions that often coexist in older adults. Their interaction may influence clinical outcomes, particularly during external stressors such as the COVID-19 pandemic. This study aimed to assess the prevalence and temporal trends of PD among hospitalized patients with T2DM in Spain (2017–2023), evaluate sex-based differences in clinical characteristics and outcomes, examine the impact of the COVID-19 pandemic, and identify predictors of PD diagnosis and in-hospital mortality (IHM). **Methods:** We conducted a retrospective, nationwide study using the Spanish National Hospital Discharge Database (RAE-CMBD). Adults aged ≥40 years hospitalized with T2DM were included. PD cases were identified using ICD-10 codes. Joinpoint regression assessed temporal trends, and multivariable logistic regression identified factors associated with PD and IHM. **Results:** Among 5.1 million T2DM-related hospitalizations, 107,931 (2.41%) involved PD. PD prevalence increased over time, particularly among women. Men accounted for most PD cases and were younger than their female counterparts. Depression and anxiety were more frequent in women and associated with PD in both sexes. IHM peaked at 14.6% in 2020, coinciding with the COVID-19 outbreak. Predictors of IHM included older age, higher comorbidity burden, dementia, and COVID-19 diagnosis. **Conclusions:** The coexistence of PD and T2DM in hospitalized patients is associated with clinical complexity and increased mortality. Personalized, multidisciplinary care is essential to address sex-specific patterns, psychiatric comorbidities, and vulnerability to systemic stressors.

## 1. Introduction

Type 2 diabetes mellitus (T2DM) and neurodegenerative diseases like Parkinson’s disease (PD) are growing public health concerns, especially in aging populations. As people live longer and lead more sedentary lives, the number of cases of both conditions continues to rise [1,2]. PD is the second most common neurodegenerative disorder in the world, and its global prevalence is expected to double by 2040 [3]. Similarly, T2DM is affecting millions worldwide and putting pressure on health systems [4,5].

Research increasingly shows a two-way relationship between T2DM and PD. People with T2DM are at a higher risk of developing PD—between 23% and 85% higher than those without diabetes [6,7,8,9]. Also, more than 15% of people with PD have T2DM [10]. T2DM can worsen motor symptoms and speed up cognitive decline in PD patients [11]. For example, over 60% of PD patients with dementia show insulin resistance, and 30% have glucose intolerance [12]. Patients who develop T2DM prior to the onset of PD experience significantly poorer clinical outcomes and an increased risk of in-hospital mortality (IHM) compared to those who develop PD first [13]. These trends reflect a broader issue—multimorbidity. More than half of older adults live with multiple chronic conditions [14,15].

Chronic low-grade systemic inflammation is increasingly recognized as a common pathophysiological denominator in both T2DM and PD. In T2DM, persistent metabolic dysfunction promotes pro-inflammatory signaling, oxidative stress, and cytokine production that contribute not only to vascular damage but also to neurodegeneration [16]. In parallel, growing evidence suggests that neuroinflammation, mediated by activated microglia and systemic immune responses, plays a central role in the progression of PD by exacerbating dopaminergic neuron loss [17,18]. The intersection of these inflammatory pathways may help explain the increased vulnerability to PD observed in individuals with T2DM and highlights the need to consider inflammation as a shared biological substrate between metabolic and neurodegenerative disorders.

Emerging evidence suggests that not only persistent hyperglycaemia but also acute glycaemic variability exacerbates oxidative stress and neuro-inflammation, potentially accelerating dopaminergic neuron loss [19,20]. In clinical terms, stabilising glucose excursions might be as important as achieving HbA1c targets in patients with T2DM who are at risk of PD. Prospective cohorts using continuous glucose monitoring and serial inflammatory markers could clarify whether high glycaemic variability independently predicts PD onset or worsens short-term hospital outcomes.

Sex differences also matter. PD is generally more common in men, but women with T2DM may face a greater increase in PD risk than men [21]. Although the disease progresses at a similar pace in both sexes, women often report more severe symptoms. This could be due to hormonal, genetic, or behavioral factors, including how symptoms are experienced and reported [22].

The COVID-19 pandemic is another factor to consider. T2DM was identified early on as a major risk factor for severe COVID-19, increasing the chance of hospitalization and death [23,24,25]. People with PD—especially those with other health conditions like heart disease or diabetes—were also more vulnerable during the pandemic [26]. The overlap between PD, T2DM, and COVID-19 may have significantly influenced hospital admissions and outcomes, and this requires careful study.

Having both PD and T2DM seems to lead to worse outcomes. These patients tend to experience a faster decline in both movement and cognitive functions [27,28]. Understanding how sex influences this dual condition is essential to developing better prevention and treatment plans. Exploring the biological and social mechanisms behind these differences will help improve care for both men and women with PD and T2DM.

Emerging evidence indicates that PD includes different motor subtypes, such as tremor-dominant and postural instability/gait difficulty phenotypes, which differ in terms of clinical progression, symptom burden, and prognosis. In parallel, atypical Parkinsonian syndromes, such as Progressive Supranuclear Palsy (PSP) and Corticobasal syndrome (CBS), constitute distinct neurodegenerative entities that also present with Parkinsonian features but follow a more aggressive clinical course. Recent studies suggest that metabolic dysfunction, particularly glycemic variability, may influence the progression of both PD and atypical Parkinsonisms. Glycemic fluctuations have been linked to accelerated cognitive decline in patients with PSP and CBS [29].

We conducted a nationwide analysis of hospitalized patients with T2DM in Spain (2017–2023) to examine the prevalence and key demographic and clinical characteristics of comorbid PD.

## 2. Materials and Methods

### 2.1. Study Design, Data Sources, Population Characteristics, and Variables

We conducted a retrospective, population-based observational study using data from the Spanish National Hospital Discharge Database (Registro de Altas del Conjunto Mínimo Básico de Datos; RAE-CMBD), which is managed by the Spanish Ministry of Health. This database includes records from public hospitals and captures more than 95% of their discharges [30]. A detailed explanation of the methodology can be found in the official RAE-CMBD handbook available on the Ministry website [31].

We analyzed anonymized hospitalization data that included patient sex, date of birth, and admission and discharge dates. The reason for discharge was also recorded (e.g., home, death, transfer to a social institution, or voluntary discharge). Each record could contain up to 20 diagnoses and procedures, coded using the Spanish version of the International Classification of Diseases, 10th Revision (ICD-10-ES). Our study focused on hospitalizations between 1 January 2017 and 31 December 2023, in patients with T2DM (ICD-10 E11.x) who also had a diagnosis of PD identified by code G20 (see Supplementary Table S1). This diagnostic code has shown good validity in administrative datasets, with positive predictive values around 70–83% and sensitivities above 70% [32,33]. The RAE-CMBD lacks clinical variables that would allow classification into PD motor subtypes, such as tremor-dominant, akinetic-rigid, or mixed forms. Consequently, our analyses refer to overall PD, and this limitation is explicitly acknowledged.

In addition to basic sociodemographic information (such as age at admission and sex), we assessed overall health status using the Charlson Comorbidity Index (CCI), excluding T2DM [34]. We also tracked specific conditions known to be associated with PD, coded individually using ICD-10: depression [35], anxiety [36], personality disorders [37], apathy [38], sleep disorders [39], pain and sensory issues [40], tremor and axial symptoms [41], suicidal behaviors [42], alcohol-related disorders [43], and neurodegenerative diseases such as Alzheimer disease (AD) and vascular dementia (VD) [44]. COVID-19 diagnoses were included from 2020 to 2023, regardless of diagnostic position (see Supplementary Table S1).

Main outcome variables included length of hospital stay (in days) (LOHS), admission to an intensive care unit (ICU), IHM, and total hospitalization costs.

### 2.2. Statistical Analysis

To estimate the annual prevalence of PD among patients with T2DM, we divided the number of hospitalizations that included both diagnoses by the total number of T2DM hospitalizations each year. We stratified these estimates by age group and sex to observe subgroup trends.

Continuous variables such as age and LOHS were summarized using mean and standard deviation, or median and interquartile range, depending on the data distribution. Categorical variables, such as sex or presence of depression, were expressed as counts and percentages. Group comparisons were made using Chi-square or Fisher’s exact tests for categorical variables, and *t*-tests or ANOVA for continuous variables, as appropriate.

A log-linear joinpoint regression model was used to identify periods of change in annual hospitalization trends for PD in T2DM patients, stratified by sex. This statistical technique analyses temporal trends by fitting the data to a series of linear segments connected at joinpoint, forming linear splines. For each segment defined by these joinpoint, the annual percentage change (APC) was calculated. The analysis began with a minimum number of joinpoint, and additional joinpoint were sequentially tested for statistical significance [45]. In the final model, each joinpoint represented a significant change in trend, and the corresponding APC was estimated using the weighted least squares method. The analysis was conducted using the Joinpoint Regression Program, version 4.0.4 (National Cancer Institute, Bethesda, Rockville, MD, USA) [46]. To identify factors associated with PD diagnosis and IHM, we used multivariable logistic regression models. These models included all variables with *p*-values below 0.10 in univariable analyses, as well as clinically relevant covariates. Results were reported as adjusted odds ratios with 95% confidence intervals. For comparisons across calendar years, continuous variables (e.g., age, CCI, LOHS, costs) were analyzed using one-way ANOVA, while categorical variables (e.g., sex, ICU admission, IHM) were compared using Chi-square tests. All tests were two-sided. A *p*-value < 0.05 was considered statistically significant.

All statistical procedures were performed using SPSS version 29.0.2.0 (IBM Corp., Armonk, NY, USA).

### 2.3. Ethics Statement

The RAE-CMBD is an anonymized administrative database. Because it contains no identifiable personal information, no ethics approval or patient consent was required. Access to the data can be requested through the Spanish Ministry of Health.

## 3. Results

### 3.1. Overall Trends

From 2017 to 2023, hospitalizations for patients with T2DM in Spain increased from 620,395 to 709,181, a 14% rise (*p* < 0.001 for trend). Within this group, the number of patients also diagnosed with PD grew from 14,287 in 2017 (2.30%) to 17,328 in 2023 (2.44%), reaching a peak of 2.51% in 2022 (*p* < 0.001) (Table 1). A year-by-year breakdown by sex is available in Supplementary Tables S2 and S3.

Men consistently represented a higher proportion of PD cases (~55–58%), and patients had a stable average age (~80.5 years). While ICU admissions and costs per stay increased, the median length of hospital stay remained constant. Notably, IHM peaked in 2020 during the COVID-19 pandemic at 14.6%.

**Figure 1:**
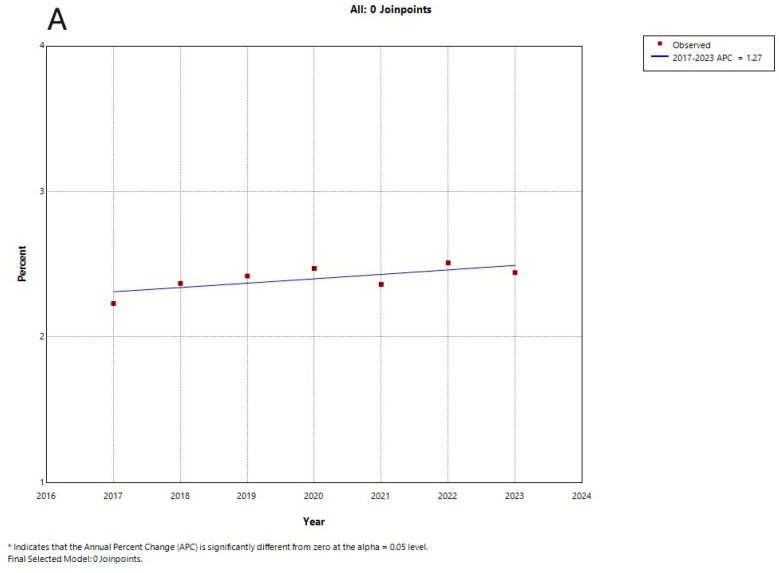

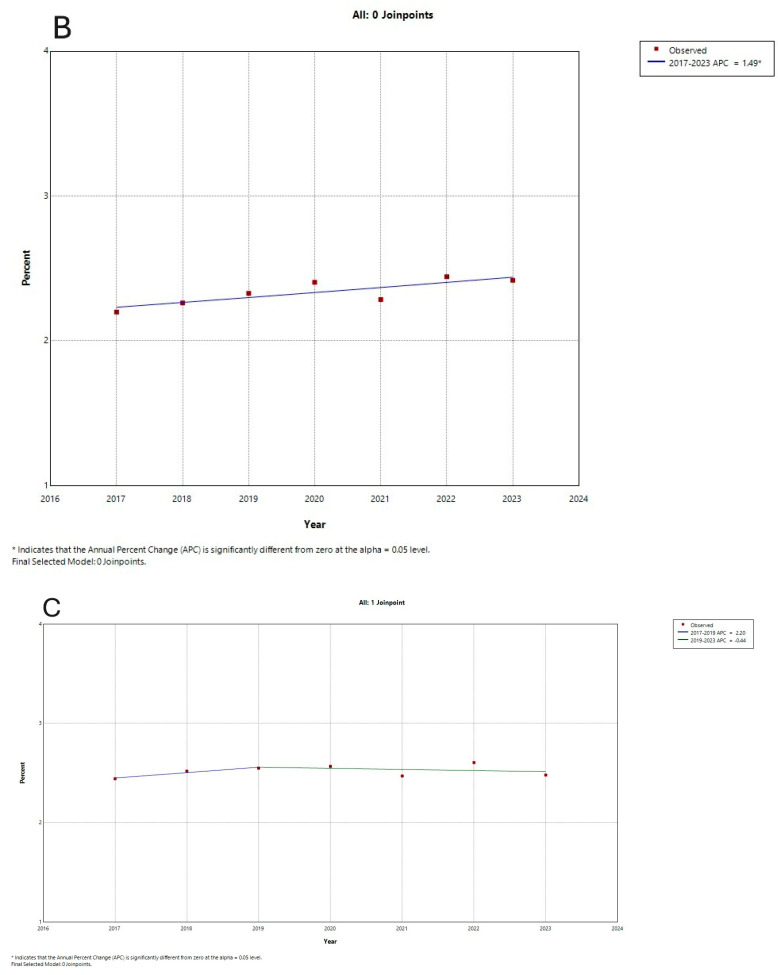
Joinpoint regression analysis of PD prevalence trends (2017−2023). Curve (**A**): total population; (**B**): men; (**C**): women.

Joinpoint regression analysis showed an upward trend in PD prevalence in the total population (Figure 1A), with an APC of +1.27%, although this was not statistically significant. Among men (Figure 1B), a significant increasing trend was observed, with an APC of +1.49% (*p* < 0.05). In women (Figure 1C), the analysis identified a significant rise between 2017 and 2019 (APC: +2.22%; *p* < 0.05), followed by a slight, non-significant decline after 2019 (APC: −0.44%) (Figure 1A–C).

The average age of PD patients remained stable over time, rising slightly from 80.3 years in 2017 to 80.5 years in 2023 (*p* < 0.001). CCI also increased slightly from 0.98 to 1.04 (*p* < 0.001). LOHS stayed consistent at 6–7 days (*p* = 0.116), but the median cost per admission rose from EUR 3560 in 2017 to EUR 4231 in 2023 (*p* < 0.001; Table 1). Additional yearly data on LOHS, IHM, ICU use, and hospital costs are presented in Supplementary Tables S2 and S3.

IHM peaked significantly in 2020, rising from 10.7% in 2019 to 14.6% (*p* < 0.001). This was followed by a gradual decline, reaching 11.4% in 2023. ICU admissions also increased slightly from 3.03% in 2017 to 3.75% in 2023 (*p* < 0.001) (Table 1).

### 3.2. Sex-Specific and Comorbidity Patterns

Among hospitalized patients with T2DM, men consistently accounted for a slight majority of PD cases, increasing from 54.9% in 2017 to 58.5% in 2023 (*p* < 0.001), whereas the proportion of diagnoses in women rose more modestly (Table 1). By 2023, the average age of female patients reached 81.9 years, compared to 79.5 years in men (Table 2 and Table 3). While the overall age of PD patients remained relatively stable during the study period, rising slightly from 80.3 to 80.5 years (*p* < 0.001), sex-specific analyses reveal nuanced clinical differences. The CCI increased slightly over time (from 0.98 to 1.04; *p* < 0.001), with PD patients having fewer severe coexisting conditions compared to non-PD patients (OR = 0.84, both sexes). LOHS remained stable at 6–7 days (*p* = 0.116), but median hospitalization costs rose from €3560 in 2017 to €4231 in 2023 (*p* < 0.001; Table 1).

Neuropsychiatric comorbidities were more frequent in women. Depression and anxiety were diagnosed in 11.95% and 8.7% of women, respectively, compared to 5.77% and 3.0% in men. Both disorders were independently associated with PD: depression had odds ratios (OR) of 1.72 in women and 2.30 in men; anxiety showed ORs of 1.37 in women and 1.58 in men (Table 4). Tobacco use, more common among men (28.1%), was linked to lower odds of PD (OR = 0.69, both sexes).

Table 2 also reveals that personality disorders, though infrequent, increased in prevalence among women (0.28% to 0.71%), as did sleep disorders (0.74% to 1.50%). The increase in axial symptoms (8.59% to 10.71%) and essential tremor (3.69% to 4.86%) suggests progressive motor deterioration. Hospitalization costs in women rose, although LOHS remained stable.

In men (Table 3), personality disorders also increased (0.34% to 0.77%), while alcohol use rose steadily to 7.19% in 2023. AD showed a subtle increase after 2020. Despite growing comorbidity indicators, LOHS remained unchanged, but hospitalization costs rose.

Dementia of any cause was present in 16.2% of women and 15.4% of men with PD in 2023. AD and VD were each found in 4–6% of cases. Predominant axial symptoms were more common in men (13.0%) than in women (10.7%), while essential tremor was slightly more frequent in women (4.9% vs. 3.8%). Other strong predictors of PD included hypoglycemia (OR = 1.24, both sexes) and axial motor symptoms (OR = 2.39, both sexes). Being male (OR = 1.39; 95% CI: 1.37–1.41) and aged ≥85 years (OR_men = 8.54; OR_women = 6.01) were also independently associated with PD (Table 4).

Among hospitalized patients with T2DM, men consistently accounted for a slight majority of PD cases, increasing from 54.9% in 2017 to 58.5% in 2023 (*p* < 0.001). Meanwhile, the proportion of PD diagnoses in women rose more modestly (Table 1). By 2023, the average age was 81.9 years in women and 79.5 years in men (Table 2 and Table 3). Neuropsychiatric disorders like depression and anxiety were more frequently diagnosed in women (11.95% and 8.7%) than in men (5.77% and 3.0%). Both conditions were independently associated with PD: depression had odds ratios of 1.72 for women and 2.30 for men, while anxiety showed ORs of 1.37 for women and 1.58 for men. Smoking—more common among men (28.1%)—was linked to lower odds of PD (OR_both sexes = 0.69) (Table 4).

In 2023, dementia of any cause affected 16.2% of women and 15.4% of men with PD. Among those with dementia, AD and VD were each present in approximately 46% of cases. 

Predominant axial symptoms were more common in men (13.0%) than in women (10.7%), while the concomitant diagnosis of essential tremor was slightly more frequent in women (4.9% vs. 3.8%). Other strong associations of PD included hypoglycemia (OR_both sexes = 1.24) and axial motor symptoms (OR_both sexes = 2.39). A higher CCI score was associated with lower odds of PD diagnosis (OR_both sexes = 0.84), suggesting that PD patients had fewer severe coexisting conditions. Being male (OR = 1.39; 95% CI: 1.37–1.41) and aged ≥85 years (OR_men = 8.54; OR_women = 6.01) were strongly independently associated with PD (Table 4).

In addition to the neuropsychiatric and motor predictors already mentioned, multivariable analysis from Table 4 revealed that pain and sensory symptoms were independently associated with PD in women (OR = 1.18), but not significantly in men. This may reflect gender differences in symptom presentation or diagnostic coding patterns.

### 3.3. Impact of COVID-19

In 2020, hospitalizations for patients with both T2DM and PD dropped to 15,124, compared to 16,064 in 2019. At the same time, IHM spiked to 14.6%, a sharp increase from 10.7% the year before (Table 1). Diagnoses of COVID-19 in this group peaked in 2022, affecting 14.0% of men and 11.8% of women, and then declined to around 6% by 2023 (Table 2 and Table 3).

Having a COVID-19 diagnosis was independently associated with a higher risk of IHM (OR_both sexes = 1.75; 95% CI:1.62–1.88), particularly during the 2020–2021 period, when IHM rates were at their highest (OR_2020 = 1.37) (Table 5).

### 3.4. Predictors of In-Hospital Mortality (IHM)

Advanced age and a higher comorbidity burden were the most significant predictors of IHM among patients with both T2DM and PD. Compared to patients aged 40–64 years, those aged 85 or older had a much higher risk of IHM (OR_both sexes = 2.45) (Table 5). Each one-point increase in CCI was linked to a higher death risk (OR_both sexes = 1.21).

Concomitant neurodegenerative diseases were also associated with poor outcomes: AD (OR = 1.42) and VD (OR = 1.18) were all significant predictors of IHM.

Interestingly, patients diagnosed with depression or anxiety had lower odds of dying in the hospital. This was especially true for anxiety, which had a protective effect (OR = 0.70). Being male was also independently linked to a slightly higher risk of IHM (OR = 1.11) (Table 5).

## 4. Discussion

Our findings confirm that the prevalence of PD among hospitalized patients with T2DM is higher in men and increases with age, aligning with previous research that reported a comparable age-related increase in PD incidence among individuals with diabetes [8,47,48]. Moreover, recent meta-analyses have supported the association between T2DM and increased PD risk, suggesting shared pathophysiological mechanisms such as mitochondrial dysfunction, neuroinflammation, and insulin resistance [49].

The present analysis shows that non-motor symptoms, including mood disorders, are more prevalent in women with PD and contribute significantly to disability and healthcare utilization. The fact that these symptoms were independently associated with PD in our analysis underscores their clinical relevance and the need for routine screening in this population [50]. These results are consistent with recent studies showing that women with PD experience a higher burden of non-motor symptoms, particularly anxiety, depression, fatigue, and autonomic dysfunction, compared to men [51]. Notably, some authors have emphasized that non-motor fluctuations are especially frequent in women, suggesting not only a greater symptom burden but also increased variability that may complicate diagnosis and clinical management [52]. Furthermore, these observed differences may not be solely attributable to biological variation but could also reflect gender-related factors, such as a higher propensity among women to report symptoms or receive differential diagnostic attention, an area that remains underexplored [53,54]. Collectively, these findings highlight the importance of incorporating sex and gender perspectives into both clinical assessment and research design in PD.

Despite increased clinical complexity, evidenced by greater comorbidity and neuropsychiatric burden, LOHS remained stable, while hospitalization costs increased. This may suggest that care efficiency has been preserved, although each admission now requires more intensive resource utilization. Similar findings have been reported in studies of multimorbidity in neurodegenerative diseases, where rising costs were observed without proportional changes in LOHS [55]. A cohort study involving over 19,000 hospitalized PD patients likewise found that cost increases were associated with disease severity and comorbid conditions, while average LOHS remained unchanged [56]. In addition, research on older adults has shown that multimorbidity is linked to more frequent hospitalizations and longer stays, with effects moderated by age and sex, particularly among women aged 75 and older [57]. These patterns highlight the importance of considering both clinical complexity and demographic factors when assessing healthcare system burden in PD.

The peak in IHM during 2020 among patients with T2DM and PD coincides with the first wave of the COVID-19 pandemic. Similar increases in mortality among PD patients with COVID-19 have been reported in the UK and Italy [58,59]. Our results are consistent with the concept that patients with neurodegenerative and metabolic comorbidities represent a highly vulnerable group during pandemics or other systemic stressors.

This nationwide study gives new information about the link between PD and T2DM in hospitalized patients in Spain. Between 2017 and 2023, we saw a slow but steady rise in PD cases among people with T2DM. This trend was mostly seen in women and backs up previous proof that T2DM makes people more likely to have neurodegenerative diseases like PD [45].

Joinpoint analysis showed an increase in PD prevalence from 2017 to 2019, and then it remained stable until 2023. This stabilization could be a result of the disruptions the COVID-19 pandemic caused to the accessibility of hospital services and specialist care. Nevertheless, even though men constituted a higher percentage of the PD cases in our cohort, the annual growth rate was higher among women; this was reflected in earlier studies and indicates a relatively higher risk for females with T2DM [21]. The women in our sample were also older and had more psychiatric comorbidities.

Mental health conditions, particularly depression and anxiety, were more common in women and were independently associated with a PD diagnosis in both sexes. This finding is consistent with previous research that highlights the comorbidity of these disorders with PD [11,34]. Surprisingly, both depression and anxiety—especially the latter—were associated with a lower risk of IHM. Similar patterns have been observed before [13], possibly due to earlier diagnosis or more consistent clinical follow-up, though diagnostic bias might also be at play.

Cognitive impairment was frequently documented. Dementia of any kind, including AD and VD, was found in a significant proportion of patients. These conditions were also among the strongest predictors of IHM [44]. This confirms the serious impact of overlapping neurodegenerative conditions on hospital outcomes and the importance of recognizing these comorbidities in routine care.

Our data also revealed sex-specific motor symptom patterns, men being more likely to have axial motor symptoms. These findings are consistent with previous reports showing clinical differences between men and women with PD [22]. In our multivariable analysis, male sex and older age—especially being 85 years or older—were strongly associated with PD. Other relevant factors included a history of hypoglycemia and the presence of axial motor symptoms. Interestingly, a higher overall comorbidity burden (measured by the CCI) was associated with lower odds of PD, which may reflect a diagnostic tendency to focus on more immediately life-threatening conditions in patients with multiple chronic illnesses. Interestingly, PD is not included in the CCI, despite its prevalence as a chronic and highly comorbid condition. This omission may also influence the paradoxical epidemiological association between the CCI and PD in the CMBD database.

The COVID-19 pandemic had a clear effect on this vulnerable population. Hospital admissions for patients with both T2DM and PD dropped sharply in 2020, while IHM rose significantly. COVID-19 infection was an independent risk factor for IHM, especially during the 2020–2021 peak [23,26]. This supports previous studies showing that individuals with T2DM and PD—particularly those with cardiovascular or metabolic comorbidities—faced higher mortality during the COVID-19 pandemic [24]. Being male and older also increased mortality risk, consistent with global findings.

Beyond classical PD, increasing evidence suggests that metabolic dysregulation, particularly T2DM and prediabetes, may also play a role in atypical Parkinsonian syndromes such as PSP and CBS. A recent study has linked glucose metabolism disturbances with cognitive decline in these conditions, indicating that glycemic variability may influence neurodegenerative progression in a broader range of Parkinsonian disorders [60]. These findings support the need to explore metabolic risk factors beyond PD and consider shared mechanisms across related pathologies.

Although our study did not assess specific T2DM treatments, recent evidence suggests that several antidiabetic medications may have neuroprotective benefits in PD. Glucagon-like peptide-1 receptor agonists (GLP-1 RAs), such as exenatide and liraglutide, have been shown in preclinical and clinical studies to restore dopaminergic neuron function, reduce neuroinflammation, and improve both motor and non-motor symptoms in PD [61,62]. Additionally, a network meta-analysis has indicated that sodium–glucose cotransporter-2 inhibitors (SGLT2 I) may lower the incidence of PD and dementia in individuals with T2DM [63]. Preclinical research further supports the neuroprotective potential of SGLT2 I through improvements in mitochondrial function and oxidative stress reduction [64]. Although evidence for metformin remains mixed, its mechanisms, such as activation of AMPK and anti-inflammatory effects, also have theoretical implications for neurodegeneration prevention [65]. Taken together, these findings highlight promising avenues for repurposing antidiabetic drugs as adjunctive or preventive therapies in PD and underline the importance of stratifying future observational and interventional studies by medication type.

Although we included several diagnostic codes related to sleep disturbances in our analysis (e.g., G47.51, F44.9, F51.9, G47.9, G47.6, R53), the database does not specify whether these were explicitly linked to REM sleep behavior disorder (RBD). Given the relevance of RBD as a prodromal marker of PD and its clinical implications [66], future research using more granular clinical data is warranted to explore these associations in greater detail [67].

This study has several limitations that should be acknowledged. First, we relied on an administrative database (RAE-CMBD), which, although comprehensive in scope, lacks detailed clinical information. As a result, we were unable to examine important factors such as the stage of PD, the duration since diagnosis, patients’ medication regimens, blood glucose levels, or socioeconomic status [68]. The database also omits clinical scales such as Hoehn–Yahr staging [69], limiting our ability to stratify analyses by disease severity, an approach that could clarify heterogeneity in outcomes. Although validation studies from Canada and Sweden report acceptable accuracy of ICD-10 codes for PD (positive predictive values 70–83% and sensitivities > 70%) [32,33], no formal validation has yet been published for the RAE-CMBD; therefore, some degree of diagnostic misclassification cannot be ruled out.

Mental health conditions and other comorbidities were identified using diagnostic codes, which likely led to underreporting. Furthermore, because our analysis was based on hospital admissions rather than on individual patients, we could not track repeat hospitalizations, something that may have introduced bias.

Furthermore, although chronic inflammation is increasingly recognized as a shared mechanism in both T2DM and PD, our study did not include inflammatory biomarkers or specific immunological variables. This limits our ability to explore the inflammatory axis as a potential contributor to the associations observed. Future studies incorporating markers of systemic or neuroinflammation are needed to better understand this pathophysiological link.

It is also important to note that our sample included only individuals aged 40 and older; therefore, the findings may not be generalizable to younger populations or those treated exclusively in outpatient settings. Additionally, diagnostic coding practices can vary between hospitals [70], and the reliability of ICD-10 codes for identifying chronic conditions like diabetes and PD may differ across healthcare environments and over time. This variability could have affected the accuracy of our case identification [60,71]. Finally, although recent studies suggest that certain antidiabetic medications, such as GLP-1 receptor agonists and SGLT2 inhibitors, may exert neuroprotective effects in PD, our dataset did not include pharmacological treatment information. Therefore, we could not explore whether specific diabetes therapies modulated the clinical characteristics or in-hospital outcomes of patients with comorbid T2DM and PD. Future research incorporating medication data is warranted to clarify these relationships.

Another limitation of the study is that the RAE-CMBD does not include clinical information on PD subtypes. As a result, we were unable to examine whether associations with T2DM differ across motor phenotypes. This is a relevant consideration, as different subtypes show distinct clinical trajectories and prognoses [72]. Tremor-dominant PD is generally associated with slower progression and fewer complications, whereas the postural instability and gait difficulty subtype tends to exhibit greater disability and poorer outcomes [73]. Subtype-specific analyses in future cohort studies could help clarify this heterogeneity.

While there are some limitations, this study also has several important strengths. It draws on seven years of data from a large, representative national cohort and uses solid statistical methods, including Joinpoint regression [46]. Our findings are consistent with previous research [11,13], which supports their reliability. Overall, this work offers meaningful insights into the burden of PD among hospitalized patients with T2DM, and it can help inform future clinical practices and public health strategies.

## 5. Conclusions

This nationwide study conducted between 2017 and 2023 provides new insights into the clinical course of hospitalized patients with concurrent T2DM and PD in Spain. Our findings reveal a modest but consistent increase in PD prevalence within this population, particularly among women, although men still represent many cases. Patients with T2DM and PD displayed distinct clinical characteristics, including older age, a lower overall comorbidity burden, and a high frequency of neuropsychiatric symptoms, especially depression and anxiety, which were more prevalent in women and independently associated with PD. Notably, although clinical complexity increased over time, length of hospital stay remained stable, while hospitalization costs rose, suggesting greater resource intensity per admission.

Cognitive impairment and dementia were common in this cohort and emerged as strong predictors of IHM. The presence of axial symptoms and a history of hypoglycemia were also independently associated with PD diagnosis. Interestingly, depression and anxiety were linked to lower odds of IHM, a pattern that may reflect earlier diagnosis, better clinical follow-up, or potential diagnostic bias. The impact of the COVID-19 pandemic was evident, with 2020 showing a significant increase in mortality, especially among older patients and those with a COVID-19 diagnosis.

Taken together, these findings suggest that patients with T2DM and comorbid PD represent a clinically complex and vulnerable group, with distinct patterns of disease expression, progression, and outcomes. A multidisciplinary, sex-sensitive approach that addresses motor and non-motor symptoms, cognitive decline, and systemic vulnerabilities is essential to improve care and reduce adverse outcomes in this growing population.

## Figures and Tables

**Table 1:** Characteristics of the patients hospitalized with type 2 diabetes suffering Parkinson’s disease according to year (2017–2023).

	2017	2018	2019	2020	2021	2022	2023	*p*
**T2DM n**	620,395	650,396	663,794	611,899	653,517	688,486	709,181	<0.001 *
**Prevalence of PD, n (%)**	14,287 (2.3)	15,412 (2.37)	16,064 (2.42)	15,124 (2.47)	15,435 (2.36)	17,281 (2.51)	17,328 (2.44)	<0.001 *
**Men, n (%)**	7837 (54.85)	8544 (55.44)	8998 (56.01)	8659 (57.25)	8787 (56.93)	9867 (57.1)	10,140 (58.52)	<0.001 *
**Women, n (%)**	6450 (45.15)	6868 (44.56)	7066 (43.99)	6465 (42.75)	6648 (43.07)	7414 (42.9)	7188 (41.48)	
**Age, mean (SD)**	80.3 (7.55)	80.34 (7.66)	80.52 (7.67)	80.45 (7.79)	80.42 (7.88)	80.69 (7.94)	80.51 (7.99)	<0.001 *
**CCI, mean (SD)**	0.98 (0.92)	0.98 (0.94)	1.01 (0.94)	0.99 (0.95)	1.06 (0.96)	1.01 (0.95)	1.04 (0.95)	<0.001 *
**Admission to ICU. n (%)**	433 (3.03)	565 (3.67)	619 (3.85)	491 (3.25)	492 (3.19)	566 (3.28)	649 (3.75)	<0.001 *
**IHM, n (%)**	1541 (10.79)	1728 (11.21)	1716 (10.68)	2209 (14.61)	1937 (12.55)	2070 (11.98)	1967 (11.35)	<0.001 *
**LOHS, Median (IQR)**	7 (7)	7 (7)	7 (7)	7 (8)	7 (7)	6 (8)	6 (7)	0.116
**Costs in EUR, Median (IQR)**	3560.4 (1855.7)	3571.5 (2097.1)	3666.4 (2285.4)	4295.8 (2398.7)	4242.1 (3019.9)	4487.4 (2543.1)	4231.5 (2664.2)	<0.001 *

CCI, Charlson Comorbidity Index; LOHS, length of hospital stay; ICU, intensive care unit; IHM, in-hospital mortality. *p*-values were calculated by one-way ANOVA for continuous variables and Chi-square tests for categorical variables (all two-sided). * *p* < 0.001; values without an asterisk are not statistically significant (*p* ≥ 0.05).

**Table 2:** Prevalence of Parkinson’s disease. distribution by age and clinical characteristics and in-hospital outcomes among females hospitalized with type 2 diabetes in Spain 2017–2023.

	2017	2018	2019	2020	2021	2022	2023	*p*
**Age, mean (SD)**	81.31 (7.35)	81.44 (7.49)	81.62 (7.49)	81.68 (7.58)	81.49 (7.74)	82.05 (7.61)	81.91 (7.64)	<0.001 *
**CCI, mean (SD)**	0.88 (0.87)	0.88 (0.9)	0.92 (0.89)	0.91 (0.89)	0.95 (0.9)	0.93 (0.91)	0.96 (0.91)	<0.001 *
**Hypoglycemia, n (%)**	76 (1.18)	115 (1.67)	124 (1.75)	121 (1.87)	122 (1.84)	151 (2.04)	137 (1.91)	0.007 *
**Depression sympt, n (%)**	785 (12.17)	773 (11.26)	827 (11.7)	739 (11.43)	815 (12.26)	838 (11.3)	859 (11.95)	0.349
**Anxiety, n (%)**	260 (4.03)	364 (5.3)	414 (5.86)	428 (6.62)	475 (7.15)	579 (7.81)	623 (8.67)	<0.001 *
**Personality disorders, n (%)**	18 (0.28)	43 (0.63)	31 (0.44)	35 (0.54)	60 (0.9)	58 (0.78)	51 (0.71)	<0.001 *
**Apathy, n (%)**	36 (0.56)	46 (0.67)	41 (0.58)	36 (0.56)	46 (0.69)	54 (0.73)	44 (0.61)	0.793
**Sleep disorders, n (%)**	48 (0.74)	63 (0.92)	79 (1.12)	60 (0.93)	101 (1.52)	114 (1.54)	108 (1.5)	<0.001 *
**Pain/sensory sympt, n (%)**	23 (0.36)	41 (0.6)	30 (0.42)	21 (0.32)	28 (0.42)	36 (0.49)	32 (0.45)	0.293
**Essential tremor, n (%)**	238 (3.69)	259 (3.77)	277 (3.92)	282 (4.36)	301 (4.53)	329 (4.44)	349 (4.86)	0.004 *
**Axial sympt, n (%)**	554 (8.59)	618 (9)	677 (9.58)	569 (8.8)	693 (10.42)	762 (10.28)	770 (10.71)	<0.001 *
**Alcohol, n (%)**	32 (0.5)	28 (0.41)	64 (0.91)	34 (0.53)	71 (1.07)	44 (0.59)	73 (1.02)	<0.001 *
**Tobacco use, n (%)**	168 (2.6)	208 (3.03)	263 (3.72)	205 (3.17)	285 (4.29)	334 (4.5)	354 (4.92)	<0.001 *
**All-cause dementia, n (%)**	1157 (17.94)	1230 (17.91)	1313 (18.58)	1120 (17.32)	1133 (17.04)	1267 (17.09)	1166 (16.22)	0.008 *
**Alzheimer’s disease, n (%)**	426 (6.6)	458 (6.67)	458 (6.48)	398 (6.16)	400 (6.02)	428 (5.77)	414 (5.76)	0.105
**Vascular dementia, n (%)**	255 (3.95)	259 (3.77)	303 (4.29)	267 (4.13)	256 (3.85)	271 (3.66)	271 (3.77)	0.467
**COVID-19, n (%)**	0 (0)	0 (0)	0 (0)	351 (5.43)	436 (6.56)	876 (11.82)	376 (5.23)	<0.001 *
**ICU, n (%)**	168 (2.6)	231 (3.36)	235 (3.33)	184 (2.85)	180 (2.71)	196 (2.64)	225 (3.13)	0.019 *
**IHM, n (%)**	654 (10.14)	772 (11.24)	729 (10.32)	899 (13.91)	824 (12.39)	916 (12.36)	812 (11.3)	<0.001 *
**LOHS, Median (IQR)**	7 (7)	7 (7)	7 (7)	7 (7)	7 (7)	6 (8)	6 (7)	0.474
**Cost EUR, Median (IQR)**	3560.45 (1865.5)	3571.53 (1910.24)	3666.44 (2169.27)	4217.24 (2547.01)	4242.09 (2900.59)	4487.4 (2575.23)	4231.47 (2664.19)	<0.001 *

CCI, Charlson Comorbidity Index; LOHS, length of hospital stay; ICU, intensive care unit; IHM, in-hospital mortality; SD, standard deviation; IQR, interquartile range. * *p* < 0.001; values without an asterisk are not statistically significant (*p* ≥ 0.05).

**Table 3:** Prevalence of Parkinson’s disease. distribution by age and clinical characteristics and in-hospital outcomes among males hospitalized with type 2 diabetes in Spain 2017–2023.

	2017	2018	2019	2020	2021	2022	2023	*p*
**Age, mean (SD)**	79.47 (7.61)	79.46 (7.68)	79.66 (7.71)	79.53 (7.81)	79.61 (7.88)	79.66 (8.03)	79.51 (8.09)	0.303
**CCI, mean (SD)**	1.06 (0.96)	1.05 (0.96)	1.08 (0.97)	1.05 (0.98)	1.13 (1)	1.08 (0.98)	1.09 (0.98)	<0.001 *
**Hypoglycemia, n (%)**	128 (1.63)	121 (1.42)	153 (1.7)	156 (1.8)	143 (1.63)	157 (1.59)	152 (1.5)	0.499
**Depression sympt, n (%)**	459 (5.86)	451 (5.28)	474 (5.27)	512 (5.91)	549 (6.25)	549 (5.56)	585 (5.77)	0.054
**Anxiety, n (%)**	124 (1.58)	158 (1.85)	175 (1.94)	184 (2.12)	218 (2.48)	293 (2.97)	302 (2.98)	<0.001 *
**Personality disorders, n (%)**	27 (0.34)	30 (0.35)	44 (0.49)	42 (0.49)	46 (0.52)	66 (0.67)	78 (0.77)	<0.001 *
**Apathy, n (%)**	48 (0.61)	67 (0.78)	82 (0.91)	78 (0.9)	76 (0.86)	89 (0.9)	72 (0.71)	0.205
**Sleep disorders, n (%)**	74 (0.94)	94 (1.1)	101 (1.12)	104 (1.2)	137 (1.56)	123 (1.25)	116 (1.14)	0.016 *
**Pain/sensory sympt, n (%)**	10 (0.13)	23 (0.27)	15 (0.17)	19 (0.22)	24 (0.27)	28 (0.28)	22 (0.22)	0.245
**Essential tremor, n (%)**	221 (2.82)	231 (2.7)	287 (3.19)	247 (2.85)	287 (3.27)	283 (2.87)	389 (3.84)	<0.001 *
**Axial symptoms, n (%)**	877 (11.19)	875 (10.24)	1043 (11.59)	1033 (11.93)	1070 (12.18)	1227 (12.44)	1319 (13.01)	<0.001 *
**Suicide Attempt, n (%)**	2 (0.03)	1 (0.01)	0 (0)	2 (0.02)	2 (0.02)	2 (0.02)	1 (0.01)	0.825
**Alcohol, n (%)**	432 (5.51)	466 (5.45)	566 (6.29)	588 (6.79)	582 (6.62)	716 (7.26)	729 (7.19)	<0.001 *
**Tobacco use, n (%)**	2042 (26.06)	2203 (25.78)	2404 (26.72)	2318 (26.77)	2420 (27.54)	2806 (28.44)	2845 (28.06)	<0.001 *
**All-cause dementia, n (%)**	1323 (16.88)	1328 (15.54)	1469 (16.33)	1437 (16.6)	1374 (15.64)	1506 (15.26)	1562 (15.4)	0.013 *
**Alzheimer’s disease, n (%)**	353 (4.5)	368 (4.31)	412 (4.58)	358 (4.13)	310 (3.53)	348 (3.53)	385 (3.8)	<0.001 *
**Vascular dementia, n (%)**	421 (5.37)	373 (4.37)	425 (4.72)	423 (4.89)	433 (4.93)	431 (4.37)	435 (4.29)	0.006 *
**COVID-19, n (%)**	0 (0)	0 (0)	0 (0)	471 (5.44)	651 (7.41)	1383 (14.02)	642 (6.33)	<0.001 *
**IHM, n (%)**	887 (11.32)	956 (11.19)	987 (10.97)	1310 (15.13)	1113 (12.67)	1154 (11.7)	1155 (11.39)	<0.001 *
**LOHS, Median (IQR)**	6 (8)	6 (8)	6 (8)	6 (8)	7 (8)	6 (7)	6 (7)	0.331
**Costs EUR, Median (IQR)**	3592.7 (1849.1)	3571.5 (2274.9)	3666.4 (2285.4)	4481.8 (2330.5)	4362.3 (2893.5)	4487.4 (2512.6)	4231.5 (2664.2)	<0.001 *

CCI, Charlson Comorbidity Index; LOHS, length of hospital stay; ICU, intensive care unit; IHM, in-hospital mortality; SD, standard deviation; IQR, interquartile range. * *p* < 0.001; values without an asterisk are not statistically significant (*p* ≥ 0.05).

**Table 4:** Multivariable analysis of the factors associated with the presence of Parkinson’s disease among men and women hospitalized with type 2 diabetes in Spain 2017–2023.

	Presence of PD		
	Men	Women	Both Sexes
	OR (95% CI)	OR (95% CI)	OR (95% CI)
**2018**	1.02 (0.99–1.05)	1.03 (0.99–1.06)	1.02 (1–1.05)
**2019**	1.04 (1.01–1.08)	1.04 (1–1.07)	1.04 (1.02–1.06)
**2020**	1.06 (1.03–1.09)	1.04 (1–1.07)	1.05 (1.02–1.07)
**2021**	1 (0.97–1.03)	1 (0.96–1.03)	1 (0.97–1.02)
**2022**	1.03 (1–1.06)	1.04 (1–1.07)	1.03 (1.01–1.06)
**2023**	1.03 (1–1.07)	0.99 (0.96–1.03)	1.02 (0.99–1.04)
**65–74 year**	3.58 (3.44–3.74)	3.35 (3.15–3.56)	3.53 (3.41–3.65)
**75–84 year**	7.79 (7.48–8.11)	6.32 (5.96–6.69)	7.35 (7.11–7.6)
**≥85 year,**	8.54 (8.19–8.91)	6.01 (5.67–6.37)	7.49 (7.24–7.75)
**CCI**	0.83 (0.83–0.84)	0.86 (0.86–0.87)	0.84 (0.84–0.85)
**Hypoglycemia, n (%)**	1.31 (1.23–1.4)	1.17 (1.09–1.25)	1.24 (1.18–1.3)
**Depression symptoms** **, n (%)**	2.3 (2.22–2.39)	1.72 (1.68–1.77)	1.93 (1.88–1.97)
**Anxiety,** **n (%)**	1.58 (1.49–1.66)	1.37 (1.32–1.42)	1.44 (1.4–1.49)
**Sleep disorders** **, n (%)**	1.21 (1.12–1.31)	1.2 (1.1–1.3)	1.21 (1.14–1.28)
**Pain and sensory symptoms** **, n (%)**	1.07 (0.9–1.27)	1.18 (1.02–1.35)	1.13 (1.01–1.26)
**Essential tremor** **, n (%)**	1.79 (1.71–1.88)	1.89 (1.8–1.98)	1.84 (1.78–1.9)
**Axial symptoms** **, n (%)**	2.62 (2.55–2.69)	2.07 (2.01–2.14)	2.39 (2.34–2.43)
**Suicide Attempt, n (%)**	0.85 (0.45–1.61)	1.49 (0.76–2.93)	1.11 (0.7–1.76)
**Alcohol, n (%)**	0.84 (0.81–0.86)	0.95 (0.85–1.06)	0.84 (0.81–0.86)
**Tobacco use, n (%)**	0.7 (0.69–0.71)	0.65 (0.61–0.68)	0.69 (0.68–0.7)
**All-cause dementia, n (%)**	2.24 (2.17–2.31)	1.72 (1.66–1.78)	1.98 (1.93–2.03)
**Alzheimer’s disease, n (%)**	0.6 (0.57–0.63)	0.65 (0.62–0.68)	0.61 (0.59–0.63)
**Vascular dementia, n (%)**	1.3 (1.24–1.36)	1.17 (1.11–1.24)	1.26 (1.21–1.3)
**COVID-19, n (%)**	1.13 (1.09–1.18)	1.03 (0.99–1.08)	1.1 (1.06–1.13)
**Gender**			1.39 (1.37–1.41)

CCI: Charlson Comorbidity Index. OR: Odds Ratio. CI: Confidence interval.

**Table 5:** Multivariable analysis of the factors associated with IHM among patients with type 2 diabetes and concomitant Parkinson’s disease, Spain 2017–2023.

	IHM of Patients with T2DM and PD		
	Men	Women	Both Sexes
	OR (95% CI)	OR (95% CI)	OR (95% CI)
**2018**	0.99 (0.9–1.09)	1.12 (1.01–1.26)	1.05 (0.97–1.13)
**2019**	0.95 (0.86–1.05)	1 (0.89–1.12)	0.97 (0.9–1.04)
**2020**	1.35 (1.23–1.48)	1.38 (1.24–1.54)	1.37 (1.27–1.47)
**2021**	1.07 (0.97–1.17)	1.19 (1.07–1.33)	1.12 (1.04–1.2)
**2022**	0.93 (0.85–1.03)	1.14 (1.02–1.27)	1.02 (0.95–1.1)
**2023**	0.96 (0.87–1.05)	1.07 (0.96–1.2)	1.01 (0.94–1.08)
**65–74 year**	1.31 (1.1–1.57)	0.92 (0.72–1.17)	1.17 (1.01–1.34)
**75–84 year**	1.78 (1.5–2.1)	1.35 (1.08–1.7)	1.62 (1.42–1.85)
**≥85 year**	2.63 (2.22–3.12)	2.09 (1.66–2.62)	2.45 (2.14–2.8)
**CCI**	1.19 (1.17–1.22)	1.24 (1.21–1.28)	1.21 (1.19–1.24)
**Hypoglycemia, n (%)**	1.03 (0.86–1.25)	1.13 (0.93–1.39)	1.08 (0.94–1.24)
**Depression symptoms** **, n (%)**	0.87 (0.78–0.98)	0.88 (0.8–0.97)	0.87 (0.81–0.94)
**Anxiety,** **n (%)**	0.66 (0.54–0.8)	0.71 (0.63–0.81)	0.7 (0.63–0.78)
**Sleep disorders** **, n (%)**	1.09 (0.88–1.35)	0.94 (0.72–1.24)	1.03 (0.87–1.22)
**Pain/sensory symptoms** **, n (%)**	0.86 (0.5–1.48)	0.62 (0.36–1.05)	0.72 (0.5–1.06)
**Essential tremor** **, n (%)**	0.84 (0.72–0.97)	0.77 (0.66–0.89)	0.8 (0.72–0.89)
**Axial symptoms** **, n (%)**	1.23 (1.14–1.32)	1.14 (1.04–1.25)	1.19 (1.13–1.26)
**Suicide Attempt, n (%)**	1 (0–0)	1 (0–0)	1 (0–0)
**Alcohol, n (%)**	1.03 (0.92–1.15)	0.63 (0.39–1.01)	1 (0.9–1.11)
**Tobacco use, n (%)**	0.77 (0.73–0.82)	0.83 (0.69–0.99)	0.78 (0.74–0.82)
**All-cause dementia, n (%)**	0.93 (0.84–1.02)	1 (0.9–1.12)	0.96 (0.9–1.03)
**Alzheimer’s dementia, n (%)**	1.45 (1.27–1.65)	1.37 (1.2–1.58)	1.42 (1.29–1.56)
**Vascular dementia, n (%)**	1.21 (1.06–1.38)	1.14 (0.98–1.33)	1.18 (1.07–1.3)
**COVID-19, n (%)**	1.81 (1.64–2)	1.66 (1.47–1.87)	1.75 (1.62–1.88)
**Gender**	0 (0–0)	0 (0–0)	1.11 (1.06–1.15)

T2DM: Type 2 diabetes. CCI: Charlson Comorbidity Index. OR: Odds Ratio. CI: Confidence interval.

## Data Availability

The data employed in this study were obtained through a formal agreement with the Spanish Ministry of Health and Social Services. This agreement explicitly restricts the sharing of these datasets with third parties and mandates their destruction upon completion of the study. Consequently, the data cannot be deposited in any public repository. Nonetheless, researchers who wish to request access to these data may do so by completing the form available at: https://www.sanidad.gob.es/estadEstudios/estadisticas/estadisticas/estMinisterio/SolicitudCMBD.htm (accessed on 16 December 2024). All other relevant data and methodological details are fully reported in the published article.

## Supplementary Materials

The following supporting information can be downloaded at: https://www.mdpi.com/article/10.3390/jcm14134679/s1, Table S1: Diagnostic and procedural codes from the ICD-10 classification system employed in this study; Table S2: Prevalence of Parkinson’s disease: distribution by age and clinical characteristics and in-hospital outcomes among patients hospitalized with type 2 diabetes in Spain 2017–2023, according to sex; Table S3: IHM among men and women hospitalized with type 2 diabetes according to selected concomitant conditions and to the presence of Parkinson’s Disease in Spain for the period 2017–2023.

## Abbreviations

T2DM	Type 2 diabetes mellitus
PD	Parkinson’s Disease
RAE-CMBD	Registry of Specialized Care Activity–Minimum Basic Data Set
ICD-10	International Classification of Diseases, Tenth Revision
CCI	Charlson Comorbidity Index
LOHS	length of hospital stays
ICU	intensive care unit
IHM	in-hospital mortality
APC	annual percent change
