# Supplementary material for: Demographic and Clinical Characteristics of Hospitalized Patients with Type 2 Diabetes Mellitus and Comorbid Parkinson’s Disease in Spain: A Nationwide Observational Study (2017–2023)
Source: J Clin Med. 2025 Jul 2;14(13):4679. doi: 10.3390/jcm14134679 (PMC12251495; doi:10.3390/jcm14134679)
Supplement: Supplementary file 1 [file jcm-14-04679-s001.zip › jcm-3693418-supplementary.pdf]

Supplementary Table 1. Diagnostic and procedural codes from the ICD-10 classification system employed in this study

CONDITION	ICD 10 CODES
**Type II Diabetes**	E11.x
**Parkinson's Disease**	G20.
**Hypoglycemia**	E10.641. E10.649
**Depression**	F32
**Anxiety Disorders**	F40-F48
**Personality Disorders**	F60
**Apathy**	R45
**Sleep Disorders**	G47.51. F44.9. F51.9. G47.9. G47.6. R53
**Pain and Sensory Symptoms**	R51-52
**Essential Tremor**	G25. R25
**Axial Symptoms**	R47. R13. R26
**Suicide Attempt**	T14.91
**Tobacco Use**	F17. Z72.0. Z87.891. T65.2
**All-cause dementia**	G31.0. G31.83. F00. F01. F02. F03. F05.1
**Alzheimer dementia**	G30.0. G30.1. G30.8. G30.9
**Vascular dementia**	F01.50. F01.51
**COVID 19**	B34.2. B97.29. U07.1

Supplementary Table 2. Prevalence of Parkinson disease. distribution by age and clinical characteristics and in-hospital outcomes among patients hospitalized with type 2 diabetes in Spain 2017-2023. according to sex

	Women	Men	p
**T2DM n**	1909653	2688015	<0.001
**2017 PD, prevalence n (%)**	6450(2.44)	7837(2.2)	<0.001
**2018 PD, prevalence n (%)**	6868(2.52)	8544(2.26)	<0.001
**2019 PD, prevalence n (%)**	7066(2.55)	8998(2.33)	<0.001
**2020 PD, prevalence n (%)**	6465(2.57)	8659(2.41)	<0.001
**2021 PD, prevalence n (%)**	6648(2.47)	8787(2.29)	<0.001
**2022 PD, prevalence n (%)**	7414(2.61)	9867(2.44)	<0.001
**2023 PD, prevalence n (%)**	7188(2.48)	10140(2.42)	0.108
**Age. mean (SD)**	81.65(7.56)	79.56(7.84)	<0.001
**40-64 year, n (%)**	1277(2.65)	2614(4.16)	<.001
**65-74 year, n (%)**	6579(13.68)	12276(19.54)	
**75-84 year, n (%)**	21363(44.41)	29822(47.46)	
**≥85 year, n (%)**	18880(39.25)	18120(28.84)	
**CCI, mean (SD)**	0.92(0.9)	1.08(0.97)	<0.001
**Hypoglycemia, n (%)**	846(1.76)	1010(1.61)	0.051
**Depression symptoms, n (%)**	5636(11.72)	3579(5.7)	<.001
**Anxiety, n (%)**	3143(6.53)	1454(2.31)	<.001
**Personality disorders, n (%)**	296(0.62)	333(0.53)	0.060
**Apathy. n (%)**	303(0.63)	512(0.81)	<.001
**Sleep disorders. n (%)**	573(1.19)	749(1.19)	0.991
**Pain and sensory symptoms. n (%)**	211(0.44)	141(0.22)	<.001
**Essential tremor, n (%)**	2035(4.23)	1945(3.1)	<.001
**Axial symptoms, n (%)**	4643(9.65)	7444(11.85)	<.001
**Suicide Attempt, n (%)**	9(0.02)	10(0.02)	0.724
**Alcohol, n (%)**	346(0.72)	4079(6.49)	<.001
**All-cause dementia, n (%)**	8386(17.43)	9999(15.91)	<.001
**AD,n (%)**	2982(6.2)	2534(4.03)	<.001
**VD, n (%)**	1882(3.91)	2941(4.68)	<.001
**Covid 19, n (%)**	2039(4.24)	3147(5.01)	<.001
**UCI, n (%)**	1419(2.95)	2396(3.81)	<.001
**IHM, n (%)**	5606(11.66)	7562(12.04)	0.052
**LOHS, Median (IQR)**	7(7)	6(8)	0.297
**Costs-€, Median (IQR)**	4004.49(2404.99)	4048.67(2494.28)	<0.001

Supplementary table 3. In-hospital mortality among men and women hospitalized with type 2 diabetes. according to selected concomitant condition and to the presence of Parkinson Disease in Spain for the period 2017-2023.

	IHM Men			IHM Women		
	Without PD	With PD	p-value	Without PD	With PD	p-value
**Age, mean (SD)**	77.98(10.23)	81.79(7.29)	<0.001	82.9(9.42)	83.94(6.99)	<0.001
**40-64 year, n (%)**	21756(3.61)	155(5.93)	0.000	8188(3.01)	86(6.73)	0.000
**65-74 year, n (%)**	44603(5.64)	1002(8.16)	0.000	18808(4.81)	435(6.61)	0.000
**75-84 year, n (%)**	68697(8.37)	3400(11.4)	0.000	49290(7.82)	2155(10.09)	0.000
**≥85 year, n (%)**	59152(14.4)	3005(16.58)	0.000	81127(14.28)	2930(15.52)	0.000
**CCI, mean (SD)**	1.56(1.02)	1.24(1.01)	<0.001	1.35(0.95)	1.11(0.94)	<0.001
**LOHS, n (%)**	7(11)	6(9)	<0.001	6(9)	6(8)	0.011
**Costs in euros, Median (IQR)**	5243.9(4198.11)	5078.99(2826.76)	<0.001	4934.08(3105.22)	4875.86(2725.52)	<0.001
**Hypoglycemia, n (%)**	3665(14.32)	133(13.17)	0.304	3280(13.45)	115(13.59)	0.905
**Depression symptoms, n (%)**	4362(7.02)	370(10.34)	0.000	9261(7.08)	574(10.18)	0.000
**Anxiety, n (%)**	2593(5.79)	113(7.77)	0.002	6003(6.13)	272(8.65)	0.000
**Personality disorders, n (%)**	385(4.82)	16(4.8)	0.988	356(4.3)	22(7.43)	0.010
**Apathy, n (%)**	1865(16.98)	71(13.87)	0.066	1008(13.34)	32(10.56)	0.162
**Sleep disorders, n (%)**	2965(15.76)	101(13.48)	0.093	2015(12.81)	62(10.82)	0.160
**Pain and sensory symptoms, n (%)**	310(5.92)	15(10.64)	0.020	369(5.52)	15(7.11)	0.321
**Essential tremor, n (%)**	2630(7.82)	203(10.44)	0.000	2711(7.68)	187(9.19)	0.013
**Axial symptoms, n (%)**	11741(11.84)	1093(14.68)	0.000	9833(12.54)	638(13.74)	0.016
**Suicide Attempt, n (%)**	19(4.04)	0(0)	0.516	4(1.43)	0(0)	0.718
**Alcohol, n (%)**	22277(7.34)	412(10.1)	0.000	1839(7.1)	19(5.49)	0.246
**Tobacco use, n (%)**	67598(6.32)	1666(9.78)	0.000	8825(4.75)	143(7.87)	0.000
**All-cause dementia, n (%)**	19074(14.28)	1403(14.03)	0.492	24206(14.06)	1160(13.83)	0.565
**Alzheimer dementia, n (%)**	7862(17.17)	426(16.81)	0.644	12688(15.8)	473(15.86)	0.922
**Vascular dementia, n (%)**	4441(14.98)	454(15.44)	0.511	4702(15.06)	285(15.14)	0.911
**Covid 19, n (%)**	15776(15.26)	601(19.1)	0.000	10765(14.67)	365(17.9)	0.000

IHM: in-hospital mortality.
